# The Effect of Silver Nanoparticles on Seasonal Change in Arctic Tundra Bacterial and Fungal Assemblages

**DOI:** 10.1371/journal.pone.0099953

**Published:** 2014-06-13

**Authors:** Niraj Kumar, Gerald R. Palmer, Vishal Shah, Virginia K. Walker

**Affiliations:** 1 Department of Biology, Queen's University, Kingston, Ontario, Canada; 2 Department of Biology, Dowling College, Oakdale, New York, United States of America; 3 School of Environmental Studies, Queen's University, Kingston, Ontario, Canada; RMIT University, Australia

## Abstract

The impact of silver nanoparticles (NPs) and microparticles (MPs) on bacterial and fungal assemblages was studied in soils collected from a low arctic site. Two different concentrations (0.066% and 6.6%) of Ag NPs and Ag MPs were tested in microcosms that were exposed to temperatures mimicking a winter to summer transition. Toxicity was monitored by differential respiration, phospholipid fatty acid analysis, polymerase chain reaction-denaturing gradient gel electrophoresis and DNA sequencing. Notwithstanding the effect of Ag MPs, nanosilver had an obvious, additional impact on the microbial community, underscoring the importance of particle size in toxicity. This impact was evidenced by levels of differential respiration in 0.066% Ag NP-treated soil that were only half that of control soils, a decrease in signature bacterial fatty acids, and changes in both richness and evenness in bacterial and fungal DNA sequence assemblages. Prominent after Ag NP-treatment were Hypocreales fungi, which increased to 70%, from only 1% of fungal sequences under control conditions. Genera within this Order known for their antioxidant properties (*Cordyceps/Isaria*) dominated the fungal assemblage after NP addition. In contrast, sequences attributed to the nitrogen-fixing Rhizobiales bacteria appeared vulnerable to Ag NP-mediated toxicity. This combination of physiological, biochemical and molecular studies clearly demonstrate that Ag NPs can severely disrupt the natural seasonal progression of tundra assemblages.

## Introduction

Over the last 25 years, both consumers and industrialists have benefited from the technological breakthroughs associated with the manufacture of nanoparticles (NPs). Due to their antimicrobial activities, silver NPs are popular in textiles, food contact surfaces, building materials, vehicle interiors, cosmetics, paints, air conditioners, and medical applications [1], [2], [3], [4]. This explains a global production of Ag NPs, estimated to be more than 500 tonnes annually [5]. These Ag NPs will enter the environment through the atmosphere, ground and surface waters, soil, as well as sewage sludge [6]. However, the prospective environmental burden is currently unknown since NPs are challenging to monitor once released [7]. We are concerned here that as in other examples of trans-global pollution such as polychlorinated biphenyls, insecticides and flame retardants [8], [9], [10], manufactured NPs including Ag NPs will appear in arctic ecosystems. Atmospheric deposition, in addition to the melting of the ice pack coincident with the ‘opening’ of the Arctic will only increase the probability of such contamination.

Some argue that the consequences of NP pollution are not necessarily worrisome since NPs aggregate and will then exhibit traditional properties of their bulk, parent materials. However, the propensity for NP agglomeration depends on the environment itself with characteristics of the soil and water determining if the relatively large reactive surfaces of the NPs aggregate or sorb to organic and inorganic materials [11]. In particular, it is known that agglomeration can be inhibited by adsorption to humic substances [12], [13]. Temperatures characteristic of high latitudes dictate that decomposition rates are low and thus the active layer of arctic soils are typically rich in organic carbon and humic acid. Such adsorbed NPs would then have a longer half-life, and thus potentially have a greater impact on soil communities in the arctic.

Previously, we examined the impact of several NPs including Ag NPs on bacterial communities derived from a high arctic site [14], but in those experiments soils were not exposed to temperatures consistent with seasonal change. Perturbations of community DNA profiles and denaturing gradient electrophoresis (DGGE) indicated that 0.066% Ag NPs were toxic, and particularly so for some plant symbiotic bacteria important for nitrogen fixation. Arctic vegetation growth is limited by nutrient availability [15] and because this region has a crucial role in the world's biogeochemical cycles, such initial experimental results were disturbing. Of course, silver is toxic in its bulk form [16], [17], [18], and thus it was important to examine the impact of silver microparticles (Ag MPs) as well as nanosilver. In addition, it was of interest to determine if lower latitude arctic soils show the same vulnerability as those previously examined from more northerly soil collections, and also to ascertain if fungal species are differentially susceptible compared to bacteria. Here we address these several outstanding concerns including the need for MP controls, seasonal thermal regimes, a distinct geographic origin for the soil, and more extensive microbial analysis in order to better evaluate the possible impact of these silver particle contaminants on arctic soil ecosystems.

## Materials and Methods

### Soil samples

Soil samples were collected near the Tundra Ecological Research Station at Daring Lake (64°52′N, 111°35′W) in Northwest Territories Canada, approximately 300 km from Yellowknife, the closest, permanent population center. The samples were collected under the license issued by Auora Research Institute, Inuvik, NWT, Canada (Licence no. 14514R). The region is located north of the tree line and underlain by permafrost. The mean diel temperature in winter is −35°C, with a shallow active layer melting during the spring with early summer temperatures reaching ∼15°C [19]. Three separate soil samples were collected (mid-August, 2009) using a sterile blade from the top 2–5 cm of the organic layer. The mean soil temperature at the time of collection was 8.4°C and the volumetric water content was 28.2%. The collection site is characterized as a birch-hummock type, dominated by dwarf shrubs (*Betula glandulosa*), sedges, ericaceous shrubs, Labrador tea, mosses and lichens [20]. Soil geochemical characteristics have been previously described [21] but include 46% carbon and 1.2% nitrogen content, pH 4.3, and 580 mg kg^−1^ dissolved organic carbon. Mean concentrations of Ag, Cu, and Mo in the soil were under the detection limit with Ag and Mo both at <2 ppm, and Cu at <5 ppm. Concentrations of other transition metals present in the soil include Mn at 15.9 ppm, Zn at 19.6 ppm, and Fe at 5392.8 ppm. These humic acid- rich soils were transported to the laboratory in insulated boxes with −20°C freezer packs, quickly composited into a single sample after removal of obvious stones and plant material including leaves and roots, while maintaining sterile technique at 4°C. The soil was then stored at −18°C until further experimentation.

### Treatment with NPs and MPs, and microcosm respiration monitoring

Ag NPs and Ag MPs were obtained in powder form from MK Nano (MK Impex Canada, Mississauga, Canada). The manufacturer's reported sizes of 20 nm (99.9% purity) for NPs and 3 µm (99.9% purity) for MPs were independently determined by assessing the average hydrodynamic Ag NP sizes [22], using dynamic light scattering (DLS) with a Malvern Zetasizer (Malvern Instruments Ltd., UK). These intensity-based hydrodynamic size distributions indicated that the larger NP particles were ∼40.2±12.5 nm (zeta potential: −11.1). Since Ag MPs could not be characterized (the instrument was limited to 0.6 nm −6 µm particle sizes), and to account for the large-size bias shown by DLS analysis, the particles were also examined with transmission electron microscopy (TEM; Hitachi H-7000, Japan). TEM analysis showed that the particles were well dispersed with heterogeneous sizes within the range reported by the manufacturer for the NPs, with the MP suspension dominated by particles averaging 7.5±1.9 µm but with some larger aggregates (10–13 µm) [22].

The uniformly mixed soil samples were divided into 6 aliquots (110 g), maintaining the temperature at 0°C by the use of a sterilized tub over a salt-ice mixture in a 4°C incubator. Two concentrations of Ag NPs, at 0.066% and 6.6% (w/w), were dispersed into two of the soil samples. To ensure thorough mixing, each soil sample was first divided into four separate aliquots and each mixed with 25% of the Ag NPs, prior to a final mixing for 15 min. For convenience these were designated as concentrations of low and high Ag NPs (NP_L_ and NP_H_, respectively). After thorough mixing, the soil was divided into three aliquots and sequentially packed into sealed polypropylene containers (218.5 ml; Qubit Systems, Kingston, Canada) prepared by washing with 70% ethanol, followed with a 95% ethanol rinse, and plugged with autoclaved glass wool. In an analogous procedure, containers with Ag MPs, 0.066% and 6.6% (w/w) were prepared and designated as low and high levels of Ag MPs (MP_L_ and MP_H_, respectively). The concentration chosen, while higher than that which would be found under natural conditions, allowed us to determine the environmental consequences under the incubation times chosen for the study. The concentration was also selected based previous reports of NP levels that would mimic ‘pollution-type’ contamination, as well as more critically test earlier results on Arctic soils [23], [24], [25]. The 100-fold higher MP treatment was chosen as an extreme concentration based on the calculation of the 100-fold higher NP surface to volume ratio than that of the MPs (0.17 nm^−1^ *vs*. 0.002 nm^−1^) to assist in determining if NP toxicity was due solely to the leaching of ionic silver. NP_H_ then served as a further control. Two types of unamended control soils were used for comparison; neither received NP or MP additions, but both were subjected to the same mixing treatment described above. One of the control soils was heat sterilized (autoclaved soil; 2×120°C for 20 min) prior to loading in the polypropylene containers, and the other did not receive heat treatment (control, untreated soil). A further ‘respiration negative’ control was prepared by packing 125 g of autoclaved white quartz sand (−50+70 mesh; Sigma-Aldrich, Oakville, Canada) into a similarly prepared polypropylene container.

To mimic the normal seasonal variation of the collected soil, all microcosms were subjected to a program of temperature changes that were modeled on the observed winter to summer transition temperatures at the soil collection site [19] and which had been previously used to examine the effect of seasonal change in these soils. Therefore, the microcosms and controls were first kept at −18°C for 14 days to mimic winter temperatures at the soil-air interface. They were then incubated at 0°C for 12 h followed by −18°C for 12 h, for 14 cycles, to model the winter to spring thaw transition, and finally placed at 15°C for the remainder of the experiment (71 days). All incubations and respiration data collection were done in the dark.

**Figure 1:**
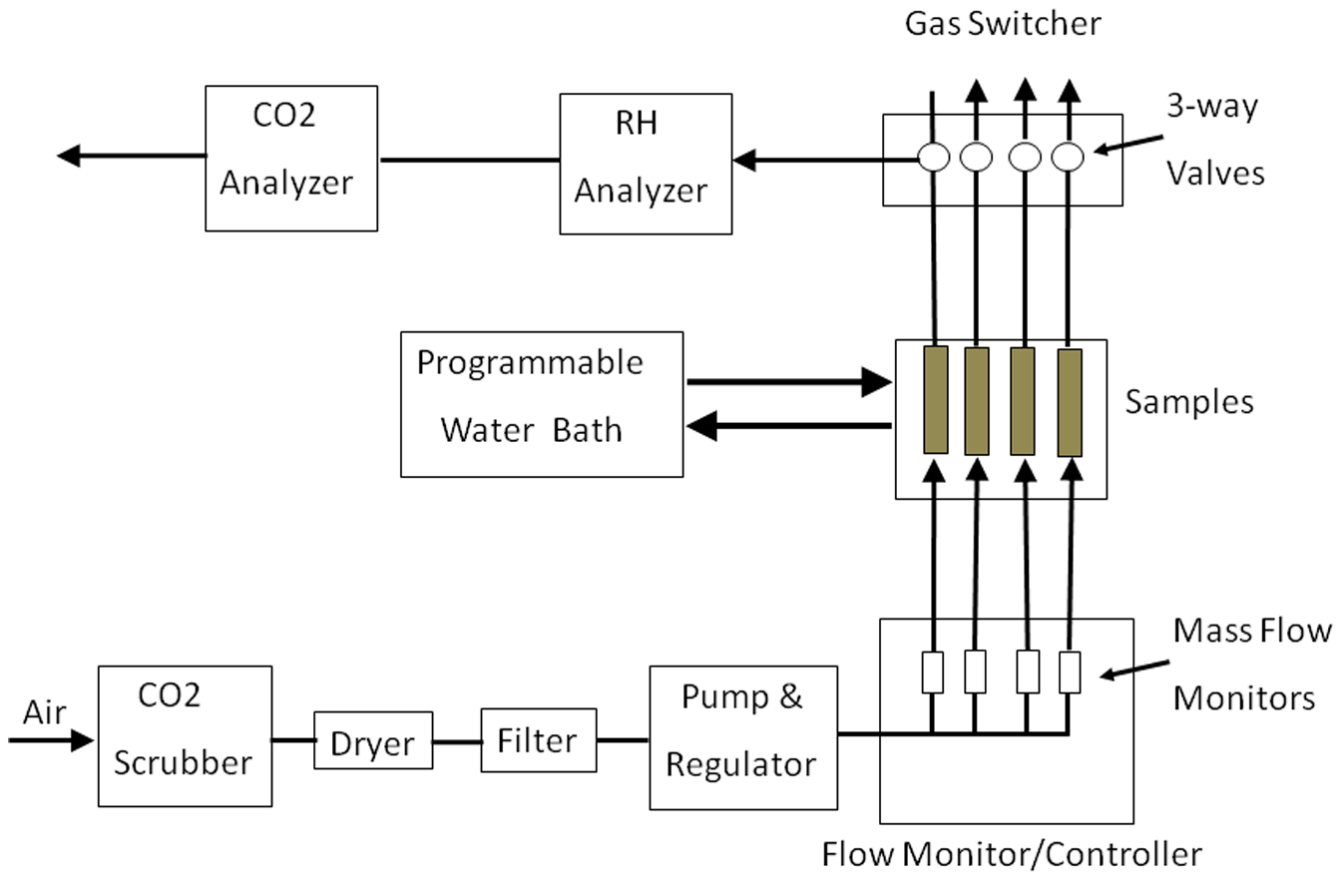
A block diagram of the multi-channel gas exchanger system (www.qubitsystems.com) that was used to assess differential respiration in the microcosms. Room air was pumped, while regulated by a gas flow controller (pump and regulator), through soda lime to remove CO_2_ (scrubber; A382), a drier (Dri-Rite; Chicago, USA), and an air filter. Flow (shown as black arrows) was regulated in each sample (shown as grey cylinders) at 50 mL min^−1^ with a flow monitor (G249 controller). Prepared soil samples were placed in sterilized 218.5 mL plastic tubes with sterile glass wool packed at both ends. Temperature was maintained with a programmable bath filled with ethylene glycol, and both temperature and humidity were monitored using sensors and a monitor (S161), as well as thermister probes (S132; not shown) fitted into ports and sealed into the container tops. Soil respiration at 1 ppm resolution was assessed using an infared CO_2_ analyzer (S151) by sampling gas with the gas sampled sequentially by three-way valves associated with a switcher (G243). Differential respiration was calculated using software (C950) that automatically subtracted the mean CO_2_ production in a particular microcosm from the autoclaved sand control (sample 1).

The temperature of the microcosms and control containers was monitored using sensors fitted into ports and sealed into the container tops, with humidity monitored using a downstream in-line sensor (Fig. 1). Pumped air, regulated by a gas flow controller (Qubit Systems) at 50 mL min^−1^ and scrubbed of CO_2_, was circulated through the chambers. Total soil respiration (1 ppm resolution) was assessed using an infared CO_2_ sensor (S151; Qubit Systems). The chamber gas was sampled sequentially with a gas switcher (G249 controller; Qubit Systems) in real time with the data captured by computer. Differential respiration was calculated automatically by the subtraction of the mean CO_2_ production in a particular microcosm from the autoclaved sand control. In order to use the equipment for other experiments, respiration from the microcosms was not continually monitored over the three months, but assessed on a near-continuous basis during the freeze-thaw cycling period and periodically after transfer to 15°C when the equipment was available, at least every 2–3 weeks throughout the incubation period. When not fitted to the respiration apparatus, the microcosms were placed in separate sealed plastic containers at the appropriate temperature. At the conclusion of the respiration monitoring, multiple replicates were obtained from different regions of the microcosms to ensure that results were representative of the treatment type, as described in the analytical sections.

### Soil fatty acid methyl ester (FAME) profiling

Triplicate soil samples were retrieved from the sequential positions (≥10 cm apart) in the microcosms using a sterile spatula. Each sample, except for the autoclaved control soil, was then sub-sampled (3 g), and placed in 15 mL plastic centrifuge tubes and shipped on ice packs to the testing facility (Keystone Labs, Edmonton, Canada). There, fatty acid methyl esters were extracted according to standard procedure of Microbial Identification System (Microbial ID Inc. [MIDI], Newark, USA) as described [26]. Only those most abundant (>1% of the chromatographic peak areas for either control or treated samples) were considered in the analysis. The fatty acid peak areas were then converted to mol% and the mean and standard errors determined. Furthermore, fatty acids were grouped into fungi, Gram-positive, Gram-negative and saturated class based on their respective indicators [27]. Unknown lipid indicators were grouped as ‘unnamed’.

### DNA extraction

The triplicate soil samples for each of the treatment group microcosms (see 2.3) were also sub-sampled (400 mg) for DNA extraction. Ethidium monoazide bromide (EMA; 100 ug/g final concentration) was added to each of the samples and after vortexing for 5 sec, they were incubated in the dark for 5 min prior to photo activation treatment, achieved by placement of the sample tubes 20 cm away from a 500 W light (400–700 nm, UL Portage Worklight; Canadian Tire, Kingston, Canada) for 60 sec. EMA is a cross-linker that reduces the probability of amplifying DNA from dead or moribund microorganisms [28], [29]. After EMA treatment, DNA was extracted from the samples using the PowerSoil DNA Isolation Kit (MO BIO Laboratories, Inc., Carlsbad, CA) as per the manufacturer's instructions.

### Denaturing gradient gel electrophoresis

Polymerase chain reaction denaturing gel electrophoresis (PCR-DGGE) was conducted by first amplifying bacterial 16S rRNA gene sequences from the extracted DNA samples (triplicates) using the primer pairs 338f (5′-ACTCCTACGGGAGGCAGCAG) with a GC clamp [30] and 907r (5′-CCGTCAATTCCTTTRAGTTT) as described by Lane *et al*. [31]. Fungal 18S rRNA gene sequences were amplified from two of the three DNA samples (duplicates) using primer pairs FF390 (5′-CGATAACGAACGAGACCT) and FR1 (5′-AICCATTCAATCGGTAIT) with a GC clamp [32]. All amplifications were performed with a Techne TC-3000 thermocycler (Barloworld Scientific, Burlington, USA). PCR reaction mixtures (total volume of 50 µL) were prepared with 0.2 mM of each dNTP, 5 µL of 10×PCR buffer, 2.0 mM MgCl_2_, 0.2 µM each primer, 1.0 U of Taq polymerase (reagents from Fermentas Canada Inc, Burlington, Canada), and 1 µL DNA template. Amplifications were started at 95°C for 10 min and followed by 35 cycles of 1 min at 95°C, 1 min at 50°C, and 1 min at 72°C (for bacterial PCRs, or 1.5 min for fungal PCRs), followed by final extension of 5 min at 72°C (for bacterial PCRs or 10 min for fungal PCRs).

The PCR products were examined by electrophoresis on 1% agarose gels, and if suitable, the amplified DNA was then separated using DGGE with a D-Code universal mutation detection system (Bio-Rad, Hercules, USA) according to manufacturer's recommendations. Bacterial amplified DNAs were separated on 6% (w/v) polyacrylamide gels with a denaturing gradient of 40 to 60% (100% denaturant contains 7 M urea and 40% formamide). The fungal PCR products were separated on 8% (w/v) gels with denaturing gradients of 25 to 55%. Equal concentrations of amplified DNA (300 ng) were loaded onto DGGE gels and electrophoresed in 1x Tris-acetate-EDTA buffer at 60°C and 65 V for 20 h (16 h for the fungal samples). Gels were subsequently stained with 1∶10,000 SYBR Green I (Invitrogen, Molecular Probes, Eugene, USA) and scanned using a ChemiGenius scanner (Syngene, Cambridge, UK). PCR-DGGE was performed multiple times on each amplified sample (triplicate samples in each treatment group for bacterial primers or duplicates for fungal primers) to ensure the reproducibility of the gel patterns. Scans of the stained gels were analyzed after rolling disc background subtraction using Quantity One image analysis software, version 4.6.7 (Bio-Rad Laboratories, Mississauga, Canada).

### Pyrosequencing and data analysis

DNA was quantified using a Nanodrop spectrophotometer (NanoDrop-1000; Ver.3.7.1; Thermo Scientific, Wilmington, USA) and used for pyrosequencing. After multiple initial PCR amplifications, all subsequent pyrosequencing procedures were performed at the Research and Testing Laboratory (Lubbock, USA). Tag-encoded FLX amplicon pyrosequencing (TEFAP) was performed as described [33], [34], [35] using the bacterium-biased primers, Gray28F (5′-TTTGATCNTGGCTCAG) and Gray519r (5′-GTNTTACNGCGGCKGCTG) and the fungus-biased primers, SSUFungiF (5′-TGGAGGGCAAGTCTGGTG) and SSUFungiR (5′-TCGGCATAGTTTATGGTTAAG). The amplified ∼500-bp fragments spanning the V1 to V3 hypervariable regions of the bacterial 16S rRNA genes and the amplified ∼400-bp fragments of the fungal 18S rRNA genes were generated using a one-step PCR with a total of 30 cycles, a mixture of Hot Start and HotStar high fidelity Taq polymerases, and amplicons originating and extending from the 28F for bacterial diversity and 515F for fungal diversity. Tag-encoded FLX amplicon pyrosequencing analyses utilized Roche 454 FLX instrumentation with Titanium reagents performed at the Research and Testing Laboratory (Lubbock, USA) based upon RTL protocols (www.researchandtesting.com). All failed sequence reads, low quality sequence ends and tags and primers were removed post-sequencing and the read collections were depleted of any non-bacterial or non-fungal ribosome sequences and chimeras using B2C2 [36], [37]. The remaining 16S and 18S rRNA sequences were denoised, assembled into clusters and queried using a distributed MegaBLAST NET algorithm [38] against the National Center for Biotechnology Information (NCBI) database. RDP ver 9 [39] and the NET and C# analysis pipeline were used for quality control and to compile, validate and further analyze the MegaBLAST outputs as described previously [37]. These were then used for sequence identity (percent of total length query sequence aligned with a given database sequence), and bacteria and fungi were classified at the appropriate taxonomic levels based upon the following criteria. Sequences with ≥95% identity to known 16S or 18S sequences were resolved at the genus level and ≥85%–<95% to the Order level. Percentages of each grouping of bacterial and fungal identifications were then individually analyzed for each of duplicate soil samples in order to obtain relative abundance information, which was then averaged. Evaluations presented at these taxonomic levels, including percentage compilations, represent all sequences resolved to their primary identification or their closest relative [37]. Data has been deposited to the NCIB BioProject Database (ID: PRJNA245847).

### Statistical analysis

The means (triplicate samples for FAME, and two independent DNA isolations used for multiple replicates of DGGE and pyrosequencing) were used for the statistical analysis. Since the assumption that the data would be normally distributed may not always hold true, nonparameteric statistical analyses were performed. Wilcoxon Matched Pair tests were used for the comparison of the mol% fatty acid profiles obtained with the FAME analysis, as well as the community composition observed at the Order level in pyrosequencing analysis between the untreated controls and the silver-treated samples. Analyses were performed using Statistica (Release 8.0) software, with a value of ≤0.05 used to indicate a significant difference between the compared values. Pyrosequencing data were further subjected to clustering analysis using Ward's method, which calculates the total sum of the squared deviations from the mean of the cluster in order to evaluate the impact of the particles on the combined microbial communities in soil [40]. Orders of bacteria and fungi present at >0.5% abundance were combined and hierarchical clustering shown as a dendogram.

## Results

### Soil respiration

**Figure 2:**
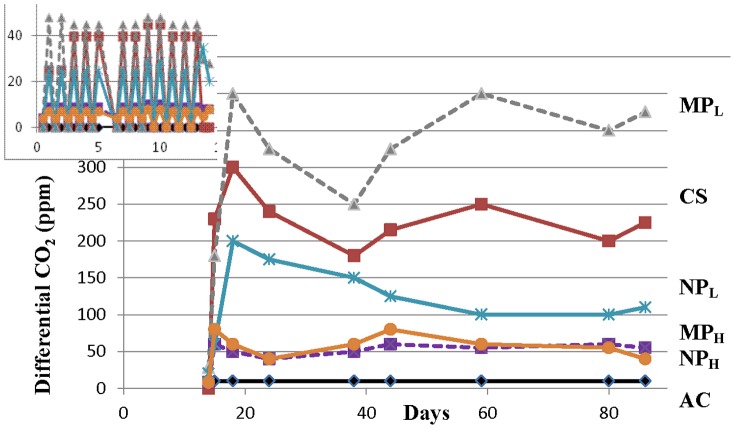
Rate of respiration in terms of differential CO_2_ (ppm) production throughout the incubation period, with the inset showing days 1–14. AC: Autoclaved soil; CS: control soil without nanoparticles or microparticles; MP_L_: soil treated with silver microparticles at 0.066% (w/v), MP_H_: soil treated with silver microparticles at 6.6% (w/v); NP_L_: soil treated with silver nanoparticles at 0.066% (w/v), and NP_H_: soil treated with silver nanoparticles at 6.6% (w/v). Incubation temperatures were alternatively cycled from −18 to 0°C (days 1–14; insert) and at 15°C (days 15–86; main graph). Autoclaved quartz sand was used as the negative control to obtain differential CO_2_.

As expected, microcosm parameters varied according to the temperature. For example, the relative humidity of the analyzed gas ranged from means of ∼30% to ∼65% at 0°C and 15°C, respectively. No differential CO_2_ was detected during ‘winter’ conditions (−18°C), but during the ‘spring’ period of 24 h cycles of −18°C to 0°C, there was a periodicity to CO_2_ discharge when soil temperatures approached 0°C (Fig. 2, insert). The detected differential CO_2_ was likely due to the release of trapped gas in the icy soil or alternatively, to low microbial respiration at 0°C. Once the microcosms were moved to the ‘summer’ temperature of 15°C, CO_2_ was detected in all of the soil samples. Autoclaved soil had a very low differential respiration of 10 ppm throughout the 71 day period at 15°C (Fig. 2). Three treatment groups showed respiration that was lower than the mean differential respiration of the untreated, control soil (230 ppm CO_2_): NP_L_, NP_H_, and MP_H_ microcosms showed mean differential CO_2_ levels of 128, 55 and 60 ppm, respectively. The MP_L_ treatment group showed the highest differential respiration at 330 ppm. At the conclusion of the respiration monitoring period there was a scattered appearance of white or beige thread-like and cottony forms resembling fungi in the MP_L_ treatment group, which showed the highest differential respiration. In contrast, there were only a few white threads in the NP_L_ -treated soil. This was not seen in the other microcosms, nor has it been observed in previous experiments where arctic soils were exposed to either temperature perturbations or metal exposure [14], [25], but not both stresses coincidently.

### Fatty acid profiling

**Figure 3:**
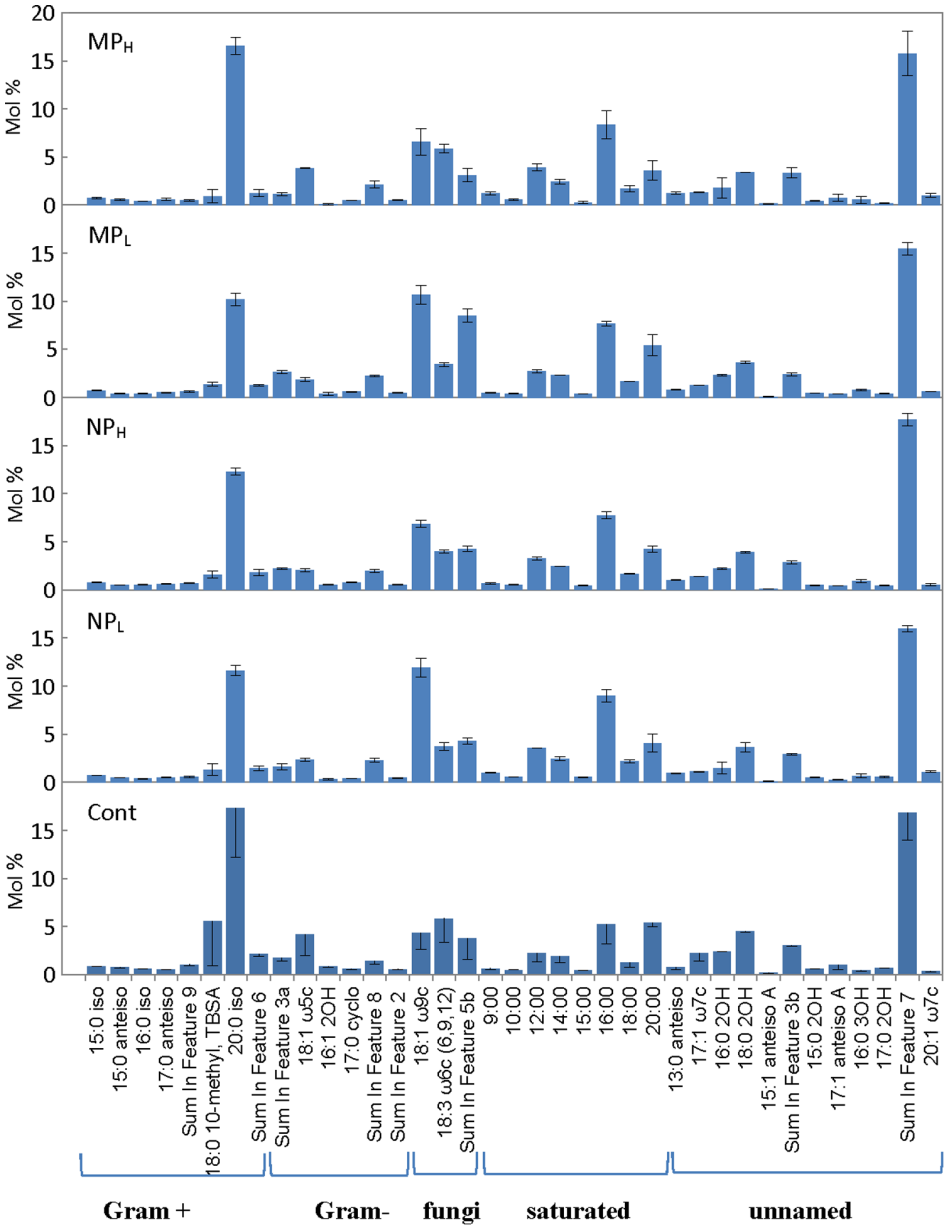
Relative amounts (mol%) of extracted fatty acids (see Methods) in control untreated (Cont) and treated soils: silver nanoparticles at 0.066% (w/v; NP_L_) and 6.6% (w/v; NP_H_), and silver microparticles at 0.066% (w/v; MP_L_) and 6.6% (w/v; MP_H_) concentrations. The bars represent the means of three independent fatty acid assessments and the standard errors. The fatty acids are grouped according to their most-frequently associated categories. “Non-signature” fatty acids are indicated as saturated or unnamed.

The FAME profiles obtained from the microcosms showed 73 different fatty acid peaks in untreated and Ag-treated soils. Of these, 37 were relatively well represented (≥0.5 mol %). In order to more easily visualize the many fatty acids in each of the treatments and controls, they were further clustered into Gram positive, Gram negative, fungal and other groups (Fig. 3). It must be noted, however, that caution must be applied when using these groupings since stress can induce changes in membrane lipids, resulting in the incorrect assignment of groupings based on such ‘signature’ fatty acids [41]. Notwithstanding this caveat, of the fatty acids attributed to Gram positive organisms, Actinomycetes (18:0 10-methyl, TBSA as a marker fatty acid) appeared to show the highest impact with silver treatments showing a decrease in relative abundance in all treatment groups (from a mean of 6.6 mol% in controls to 1.8%, 2.2%, 1.9% and 1.4% in NP_L_, NP_H_, MP_L_ and MP_H_ microcosms, respectively). Indeed, the relative abundance of many Gram positive indicators (see Fig. 3) was reduced from a total of 29 mol% in controls to 17%, 19%, 16% and 22% after treatment (in NP_L_, NP_H_, MP_L_ and MP_H_ microcosms, respectively). When compared to control soil, NP_L_ and MP treatments resulted in statistically significant reductions in the Gram positive fatty acid peak profiles (p≤0.05). Signatures for Gram negative bacteria appeared to decrease in all the treatment groups, but this was not statistically significant.

The mol% reduction in many of the bacterial signature fatty acids was inversely correlated with an apparent increase in fungal fatty acid markers in all treatment groups, irrespective of silver particle size and concentration (Fig. 3). The increase in one fungal signature fatty acid, 18:3 ω6c (6,9,12) was particularly striking in the NP_L_ and MP_L_ treatment groups since it doubled (12 and 11 mol% from 4.5 mol% in controls). With the knowledge that the statistical comparison of fungi fatty acids profile in different samples must be carried out with caution as there are only three fatty acids assigned to the group, no differences were observed for any treatments when compared to control (p > 0.05).

### Community DNA analysis

#### Bacterial communities

**Figure 4:**
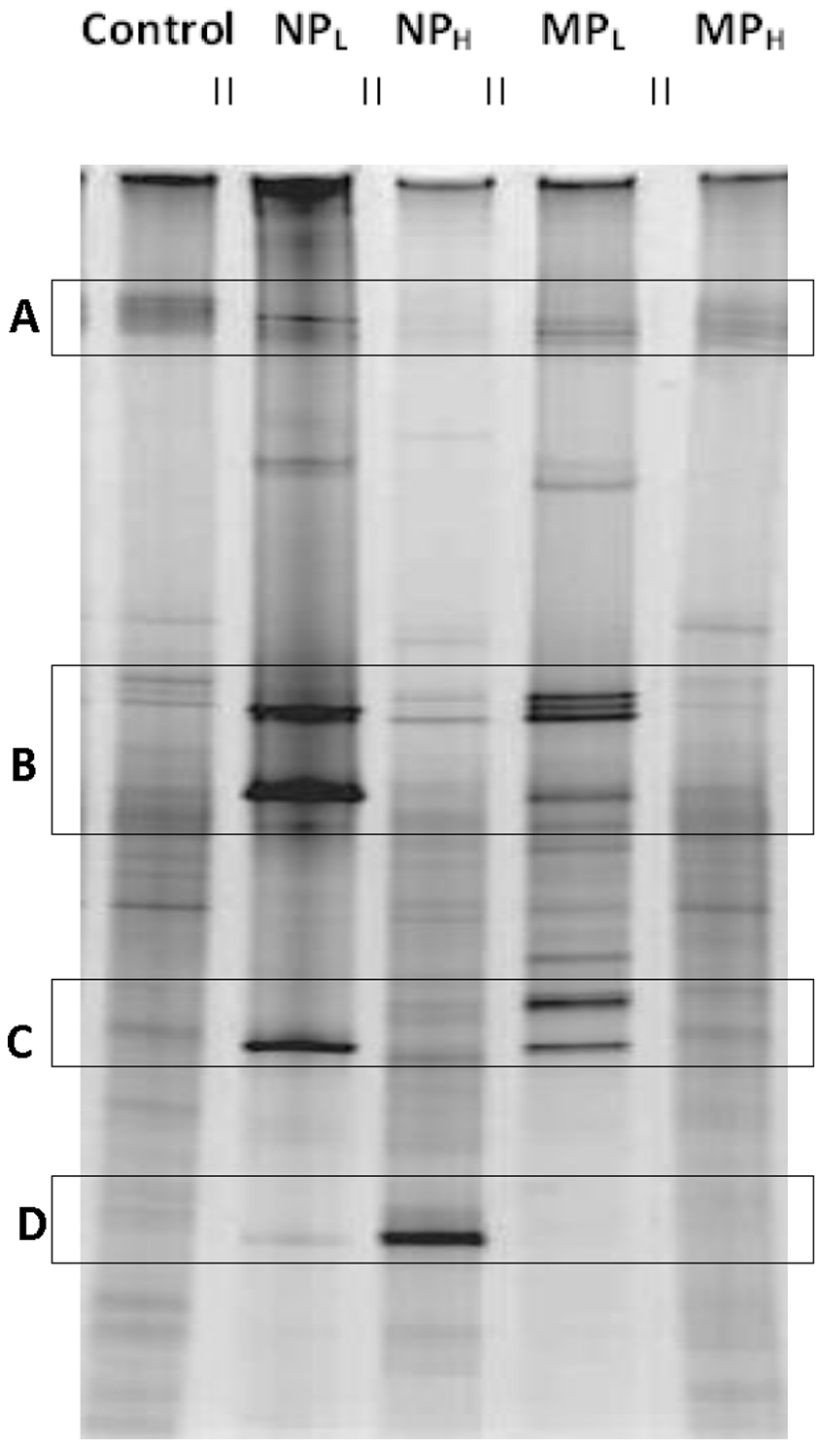
PCR-DGGE profiles of control untreated soil (CS) and treated: silver nanoparticles at 0.066% (w/v; NP_L_) and 6.6% (w/v; NP_H_), and silver microparticles at 0.066% (w/v; MP_L_) and 6.6% (w/v; MP_H_) concentrations. Bands represent the migrating positions of the amplified portions of the bacterial 16S rRNA gene sequences. Brighter and lighter bands are highlighted by boxes A, B, C and D. Gels (3–6) were electrophoresed with samples obtained from the triplicate treatment samples, but single lanes for each sample are shown in the image in order to facilitate comparisons across treatment groups. Parallel lines on the top of the gel indicate that replicate lanes were trimmed from the representative image.

The impact of Ag NPs and Ag MPs on bacterial assemblages was assessed by PCR-DGGE using bacterial primers that amplified the V3 region of 16S rRNA genes. Overall, while the number of bands was relatively similar in every DGGE profile of all the soil samples (30 for control soil *vs*. 28 NP_L_, 30 NP_H_, 32 MP_L_, 27 MP_H_), some of the microcosms showed different banding patterns (Fig. 4). Independently isolated DNA samples from the same treatment group showed the same patterns and thus a representative gel of the treatments (with duplicate or triplicate lanes digitally eliminated) is shown in order to facilitate comparisons. Generally, compared to control soil samples, NP treatments appeared to have a greater impact on the banding pattern than MPs. This is exemplified by the observation that regardless of the concentration, the addition of Ag NPs, resulted in the loss of some minor bands and an increase in the intensity of minor bands, so that they then dominated when compared to controls (boxes A to D, Fig. 4). In addition, banding patterns in control and MP_H_ microcosms appeared to be similar.

**Figure 5:**
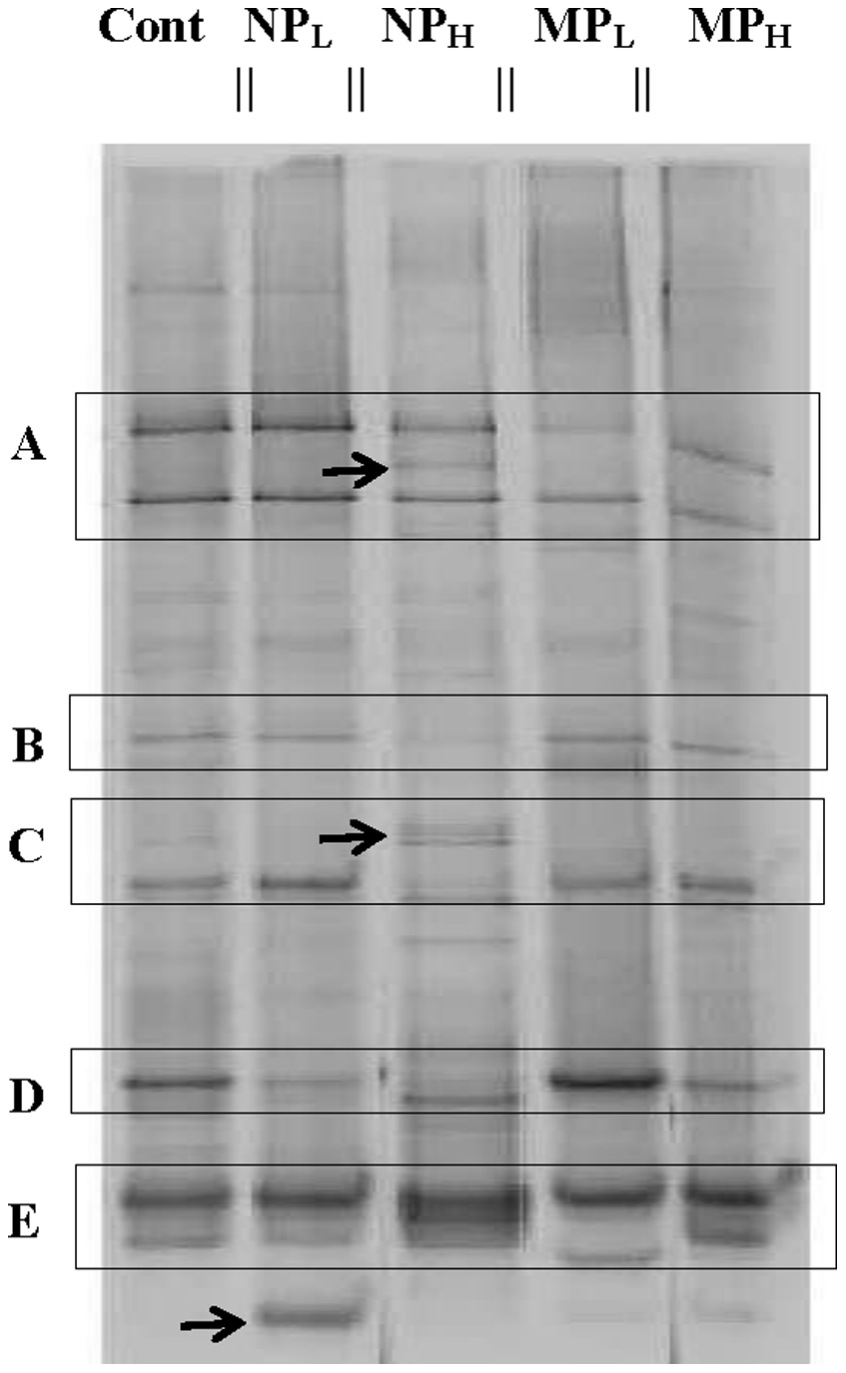
PCR-DGGE profiles of control untreated soil (CS) and treated: silver nanoparticles at 0.066% (w/v; NP_L_) and 6.6% (w/v; NP_H_), and silver microparticles at 0.066% (w/v; MP_L_) and 6.6% (w/v; MP_H_) concentrations. Bands represent the migrating positions of the amplified portions of the fungal 18S rRNA gene sequences. Brighter and lighter bands are highlighted by boxes A, B, C and D to facilitate comparisons. New bands, not shared by other treatment groups are indicated by arrows. Gels (3) were electrophoresed with samples obtained from the duplicate treatment samples, but single lanes for each sample are shown in the image in order to facilitate comparisons across treatment groups. Parallel lines on the top of the gel indicate that replicate lanes were trimmed from the representative image.

When PCR-DGGE was performed after amplification of 18S rRNA sequences a large number of major and minor bands were observed (average of 30 bands in each lane). As with the 16S rRNA gene sequence amplifications, gels were identical for the two independent soil samples analyzed for each microcosm (not shown). The majority of the dominant bands (boxes A to E; Fig. 5) in treatment groups remained unaffected as compared to controls, irrespective of the type and concentration of silver particle addition. However, some minor bands (*eg*. DNA that migrates between the A and B bands) showed changes in staining intensity, particularly in the NP_L_ treatment group.

**Figure 6:**
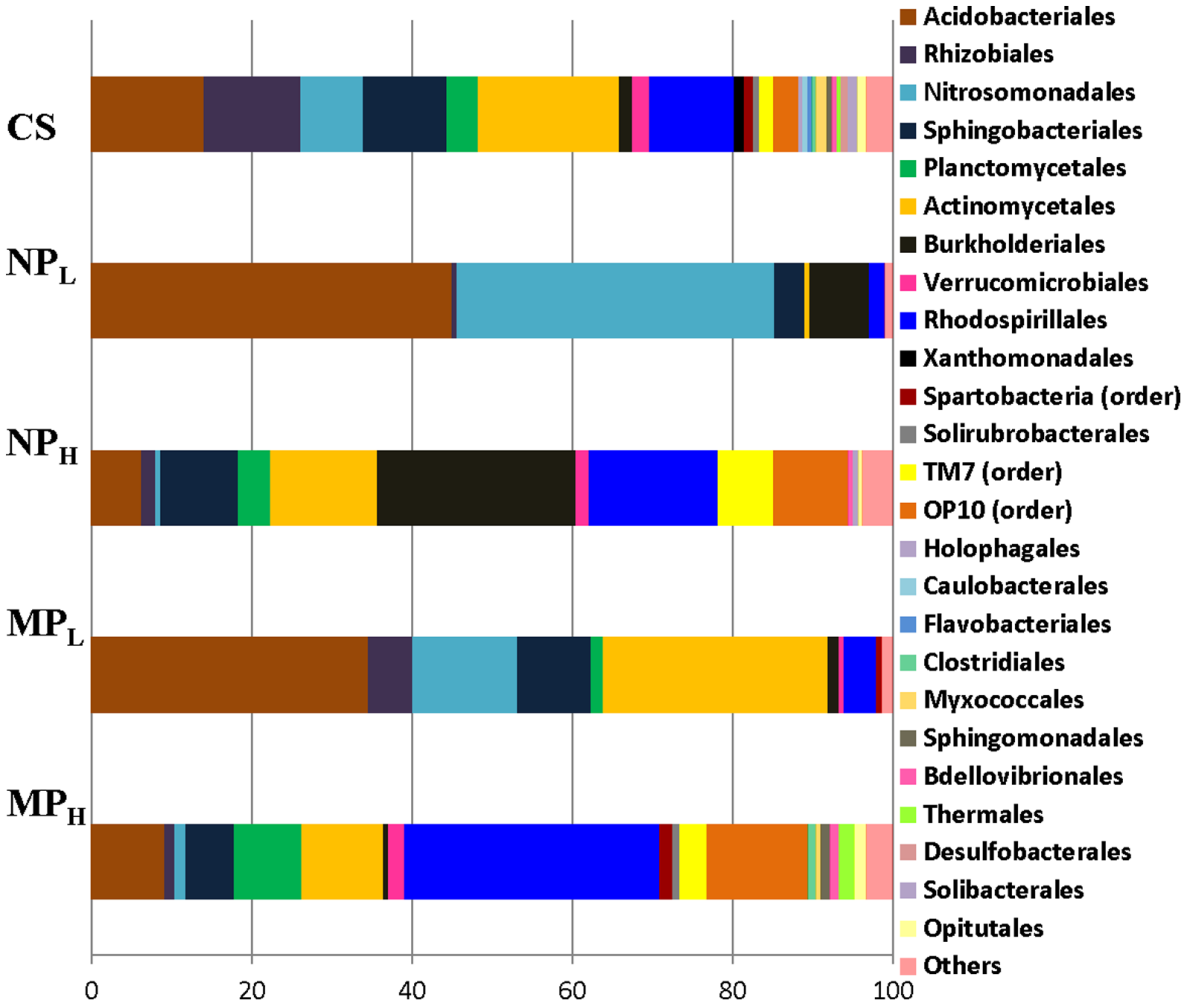
Bacterial phylogenetic composition within the Daring Lake soil at the Order level. Samples include soil that had been incubated for 86 days without NPs or MPs (Cont), soil with 0.066% NPs (NP_L_), soil incubated with 6.6% NPs (NP_H_), soil with 0.066% MPs (MP_L_) and soil with 6.6% MPs (MP_H_). Sequence identity was determined after pyrosequencing of the partial 16S rRNA genes, classified into Orders, and the means of duplicate, replicate samples of those with >0.5% abundance (for either treatment or control groups) presented in separate categories, with groupings of less abundant Orders shown as ‘Others’.

Since FAME and DGGE analysis indicated some changes in the soil community, pyrosequencing analysis of duplicate samples for each treatment group was carried out to sample the structure of the microbial assemblages. For the bacterial 16S RNA sequences identified to Order, only those present at ≥ 0.5% abundance were used, with the rest of grouped together as a single “other Orders” group. In all treatments this last category was a minor contributor to the totals. Bacteria from untreated control soil were grouped into 26 different Orders (including the “other” category). There was a reduction in total number of Orders after treating microcosms with high concentrations of silver, with bacteria in NP_H_ and MP_H_ treatments grouped into 14 and 19 Orders, respectively. In comparison, there was a significant reduction (p≤0.05) in the number of bacterial Orders after treatment with low concentrations of silver, with bacteria from NP_L_ and MP_L_ microcosms grouped into 7 and 10 Orders, respectively (Fig. 6).

In control soil, the most dominant bacterial Orders included Actinomycetales (18%), Acidobacteriales (14%), Rhizobiales (12%), Rhodospirillales (11%), and Sphingobacteriales (10%). These abundant Orders, were also represented in the MP_H_ and NP_H_ microcosms, including Actinomycetales (10% and 13%), Acidobacteriales (9% and 6%), Rhizobiales (1.2% and 1.7%), Rhodospirillales (32% and 16%), and Sphingobacteriales (6% and 10%). These changes were statistically significant (*p* = 0.009 and 0.007, for MP_H_ and NP_H_, respectively, *vs*. the untreated control). Together these 5 Orders made up 65% of the assemblage in control, 58% in MP_H_ and 47% in NP_H_ microcosms, again suggesting at best a rather modest impact of highly concentrated silver on community richness (supported by *p* values of 0.38 and 0.052 for MP_H_ and NP_H_ treatments, respectively *vs*. controls). In contrast, more obvious changes were seen at lower silver concentrations. Relative to the untreated control, MP_L_ bacterial assemblages showed increases in Actinomycetales (28%), Acidobacteriales (34%) and Burkholderiales (25%) accounting for 87% of the sequences, along with decreases in Rhizobiales (6%), Rhodospirillales (4%), and Sphingobacteriales (9%). Even more striking changes were seen after NP_L_ treatment. Only three Orders, Acidobacteriales (45%), Nitrosomandales (40%), and Burkholderiales (7.4%), with only the first as relatively abundant in the untreated control (at 14% *vs*. 7.8% and 1.6% for the other two, respectively), accounted for more than 92% of the assemblage. We investigated these changes at a deeper taxonomic level to better understand the community shifts. More than 200 genera could be identified in untreated controls, with only three genera dominating at ∼10% each (Acidobacterium, Nitrosovibrio and Rhizobium; Fig S1). Soils incubated with the lower concentration of silver retained a high abundance of these genera, with the notable exception of *Rhizobium* (decreased from 11% in controls to 2% and 0.03% in MP_L_ and NP_L_, respectively). However, the genera that rose to predominance in the MP_L_ and NP_L_ microcosms were *Burkholderia* (34%) and *Janthinobacterium* (55%), respectively.

#### Fungal communities and combined microbial orders

**Figure 7:**
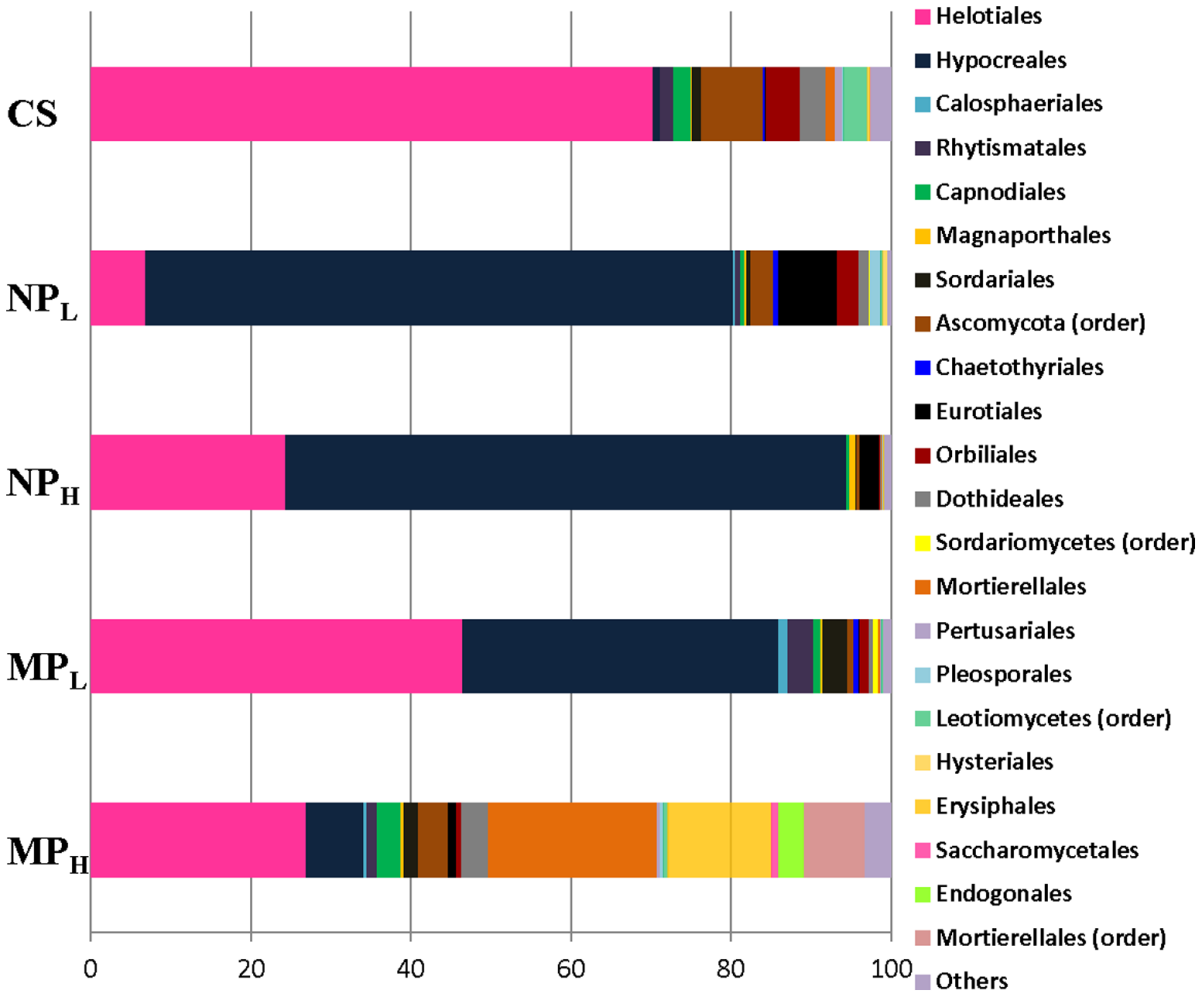
Fungal phylogenetic composition within the Daring Lake soil at the Order level. Samples include soil that had been incubated for 86 days without NPs or MPs (Cont), soil incubated with 0.066% NPs (NP_L_), soil with 6.6% NPs (NP_H_), soil with 0.066% MPs (MP_L_) and soil with 6.6% MPs (MP_H_). Sequence identity was determined after pyrosequencing of the partial 18S rRNA genes, classified into genus, and the means of duplicate, replicate samples of those with >0.5% abundance (for either treatment or control groups) presented in separate categories, with groupings of less abundant Orders shown as ‘Others’.

Similar analysis after pyrosequencing of the 18S rRNA sequences in duplicate treatment groups was performed to determine the relative abundance of the fungal sequences in the microcosms. When 18S rDNA gene sequences representing ≥0.5% of the total abundance were used for comparison, the number of Orders represented in each of the samples was similar (21 in controls and 17–22 in the treatment groups), but there were some differences in evenness depending on the treatment (Fig. 7). Control soil was dominated by Helotiales, sac fungi that often associate with dwarf shrubs in northern latitudes. These accounted for 75% of the fungal sequences. In MP_L_, MP_H_, and NP_H_ treatment groups Helotiales decreased but remained a dominant Order, representing 46%, 27% and 24% of the total abundance, respectively. However, after NP_L_ treatment, the number of reads in this Order fell from 75% in control to 7%. Another Order, Hypocreales, a minor contributor in the original fungal assemblage, increased 70-fold after silver NP treatment (1% in control soil *vs*.73% and 70% in NP_L_ and NP_H_), showing almost an inverse relationship. Statistically, the NP_H_-treatment group showed significant changes in the fungal community compared to controls (*p*≤0.05).

When analyzed at the genus level, Helotiales itself was dominated by the genus *Chalara*, a telomomorph and a litter saprotroph [42], in control and both MP-treated soils (52–93% of the sequences in that Order), but after NP_H_ or NP_L_-treatment, *Chalara* sequences dropped to 5% and 3%, respectively (Fig. S2). As noted, the abundance of Hypocreales increased after NP treatment, and the most abundant representatives of this Order were *Cordyceps* and *Isaria* (*Cordyceps* is the teleomorph of *Isaria* the anamorph) in NP_L_, and *Cordyceps* in NP_H_ treated soil (Fig. S2). Indeed, control and treatment microcosms displayed similar levels of richness with the same number of genera (34–36), but the relative proportion of these changed depending on the treatment. For example, control soils were dominated by *Chalara* (45%), NP_L_-treatment by *Cordyceps* (27%) and *Isaria* (19%) as indicated above, NP_H_ by *Cordyceps* (60%), MP_L_ soil by *Chalara* (43%), and MP_H_ treated soil by *Mortierella* (29%) and *Chalara* (15%).

**Figure 8:**
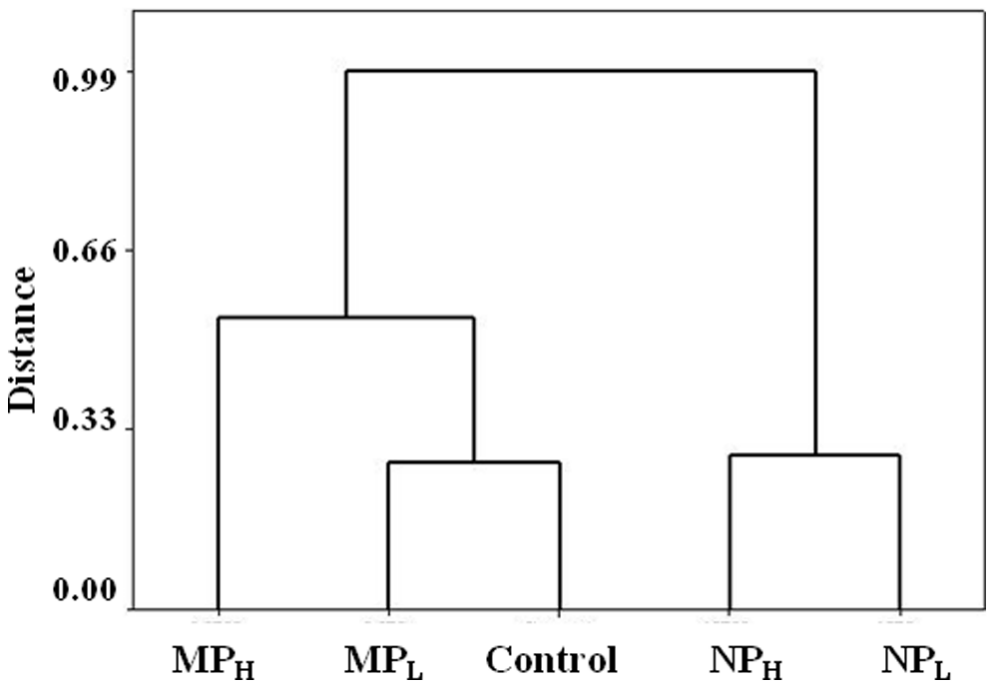
A hierarchical clustering dendogram obtained using Ward's method, an agglomerative clustering algorithm. Phylogenic composition at the Order level for both bacteria and fungi (present at >0.5% abundance) were subjected to clustering analysis, calculating the total sum of the squared deviations from the mean of the cluster. Clusters collapsed into treatment-groups irrespective of the microbe type. Treatment groups had been incubated for 86 days without NPs or MPs treatment (Control), soil incubated with 0.066% NPs (NP_L_), soil with 6.6% NPs (NP_H_), soil with 0.066% MPs (MP_L_) and soil with 6.6% MPs (MP_H_).

In order to compare the impact of the two concentrations of NPs and MPs on the bacterial and fungal Orders, Ward's method was used for statistical cluster analysis. A hierarchical tree (Fig. 8) clustered bacteria and fungi together when in the same treatment group with increasingly dissimilar clusters merging as the cluster fusion process continued. Thus microbial consortia exposed to MPs have higher degree of similarity to those Orders in control soil as compared to NP-exposed soil. The MP_L_ treatment group had a branch point closest to the control, with NP_L_ and NP_H_-treatment groups the most distant, irrespective of microbe type.

## Discussion

### Impact of silver and temperature stresses on the bacterial and fungal communities

Although we acknowledge that microcosms can only approximate natural conditions, our goal was to subject the treatment groups to a temperature regime which was similar to that found in their home environment. By itself, storage at −18°C followed by multiple freeze-thaw cycles does not change the overall evenness or richness of these sampled soils [19]. Therefore, frozen soil was subjected to freeze-thaw cycles typical of the ‘winter-summer’ transition followed by ‘summer’ incubation at 15°C, all the while exposed to silver NPs or MPs (Fig. 2). Arctic and alpine soil microbial communities change seasonally: bacterial species decrease in winter resulting in a greater proportion of fungi, and when the soils warm bacterial species again increase [43], [44], [45]. Consistent with these known seasonal changes, control soil showed a relatively higher proportion of signature fatty acid markers for bacteria compared to those associated with fungi at the end of the ‘summer’ period (Fig. 3). The addition of silver particles, however, appeared to differentially impact bacteria and there was an increase in fungal fatty acid markers, particularly the 18:1 ω 9c lipid biomarker (which more than doubled in NP_L_ and MP_L_ treatments). Fungal PCR-DGGE analysis supports this interpretation; there were few silver-mediated changes in richness, with the obvious exception of the appearance of a new, prominent band in the NP_L_ microcosm. Pyrosequencing of the amplified fungal DNA was also concordant, confirming that silver addition (except for NP_L_) did not have a large impact on community richness. For example, the sac fungi, Helotiales, a dominant Order in the control soil, remained so in the NP_H_, MP_H_ and MP_L_ treatment groups (Fig 7). When analyzed to the level of genera, control soil was dominated by representatives of Helotiales, with the litter saprotroph, *Chalara*, predominant and remaining as a major species in high silver treatment groups.

We speculate that silver particles and any associated ions inhibited bacterial division [18] and at the lower Ag concentrations, allowed fungal species to take advantage of the available resources. Therefore, in the presence of silver (NP_L_ and MP_L_), bacterial populations were not able to follow their normal temporal increase during ‘summer’ conditions, resulting in the apparent decrease in bacterial fatty acid signatures, as well as the marked reduction in bacterial Orders that dominated control soil.

Notwithstanding any differential effect on the bacterial and fungal communities, the addition of high concentrations of silver had an obvious impact on soil physiological function. Differential respiration was depressed to only ∼25% of that seen in the control soils (Fig. 2), and presumably resulted in reduced cell division [46]. Ironically, community richness of the NP_H_ and MP_H_–treatment groups appeared comparable to control soils. It is likely that many of the microbes, with the exception perhaps of some fungi, could not divide when exposed to such high silver concentrations, with the consequence that the microcosms largely retained their previous ‘summer’ DNA profiles. Since experimental protocols that are designed to assay microbial richness such as pyrosequencing do not normally allow an assessment of absolute numbers, estimation of an overall reduction in the community members may not be easily obtained. For example, the most abundant bacteria in control soil, making up 65% of the sequences, also represented the majority of Orders in NP_H_ and MP_H_ treatment groups at 47% and 58%, respectively. A further example of sac fungal predominance after high silver treatment has been already mentioned. The results of the biochemical analyses of these highly contaminated soils underscores the importance of some physiological monitoring for these types of studies, with respiration assays offering a convenient and real-time assessment of the health of microcosms.

As suggested, when bacteria were exposed to the lower silver concentration simultaneously with freeze-thaw conditions followed by a ‘summer’ period, they were unable to reach levels seen in control soil. Particularly susceptible were the Gram positive bacteria (calculated separately from Actinomycetes) from the NP_L_ and MP_L_ treatment groups; fatty acid signatures showed a similar ∼35% reduction and pyrosequencing showed an identical 95% reduction for the two soils. The fatty acid signature associated with Actinomycetes, which are filamentous, rhizosphere-associating bacteria, suggested a 66–80% reduction in all treatment groups. Pyrosequencing confirmed a reduction in the sequence reads attributable to this Order in all but the MP_L_ treatments, with decreases ranging from 18% (NP_H_) to more than 96% (NP_L_). Previously Suutari and Laakso [41] advised caution in the strict interpretation of fatty acid signatures under conditions of stress, and this work further confirms their observations. It has been suggested that the thicker peptidoglycan layer of Gram positive species may make them less susceptible to silver toxicity [47], [14], however, our results likely reflect the situation that in contrast to other microbes, these bacteria are relatively inactive during the winter season [48]. They could then succumb to the combination of stresses of the ‘winter to summer’ temperature transition combined with the silver treatments used here.

### Impact of nanoparticles on the bacterial and fungal communities

As indicted, there appeared to be a modest effect of the addition of silver (MP_L_) to the bacterial assemblage (as opposed to its impact in nanosilver form). For example, pyrosequencing showed that Orders dominating the untreated controls (65%) similarly made up the great proportion (81%) of those after MP_L_ treatment. Clustering analysis supported these observations and showed that NP-exposed microbial communities were different than those in control and MP-containing soil (Fig. 8). Other assays also indicated that the effect of Ag NP addition was striking. Differential respiration was half that of control soils, and only double that of soils treated with 100-times the silver concentration (Fig. 2). In addition, PCR-DGGE profiles showed a loss of minor bacterial bands and an increased intensity of other bacterial and fungal bands (Fig. 3 and 4). A reduction in diversity in the presence of toxins can be due to selective growth or enrichment of dominant or resistant species [49], [50], and this interpretation seems to be applicable here.

The differences in the NP_L_ DGGE banding patterns were also reflected in pyrosequencing results that showed changes in both the richness and evenness of the sequences, and which diverged from that of controls more than in any other treatment group. In particular, there was a conspicuous impact on Nitrosomonadales as well as Rhizobiales (Fig. 6). NP_L_ treatment resulted in a 5-fold increase in Nitrosomonadales sequence reads over the control, and this increase was coincident with a reduction in Rhizobiales sequences from 12% in control and 6% in MP_L_, to ≤0.7% of the sequences after NP_L_ treatment. At the genus level the impact was even more conspicuous with the number of sequences attributed to *Rhizobium* reduced 5.5-fold in MP_L_ and 370-fold in NP_L_ soil. Previous reports have also shown that Rhizobiales, representing many beneficial plant-associating bacteria, are sensitive to silver treatment including Ag NPs [51], [52], [14]. The consequences of long-term impact of nanosilver contamination on soil processes could be of great concern. For example, a dramatic decrease in nitrogen-fixing Rhizobia, along with the observed increase in ammonia oxidizing Nitrosomonadales, could result in decreased soil nitrogen over the long term. The resulting low ammonia concentration could then decrease the pH as well as lead to reduced soil fertility in a region that is already well known to be nutrient limited.

Of the 25 different bacterial Orders (≥1%) in control soil, less than a third were recovered after NP_L_ treatment. Of the most prominent in controls only the Acidobacteriales remained dominant in NP_L_. This Order along with Nitrosomonades and Burkholderiales together represented >92% of the bacterial sequences. We can only speculate on the apparent mechanisms of Ag NP resistance [53], [54], however, it is known that representatives of the Acidobacteriales can use metal as electron acceptors [55]. Although a minor genus in control (0.2%), *Janthinobacterium* (Burkholderiales) came to completely dominate (55%) all genera in the NP_L_ treatment group. Species in this genus are notable not only for their antifungal properties but for their heavy metal resistance [56] with a silver efflux gene sequence annotated in the genome (http://www.uniprot.org/uniprot/A6SYR2). *Pseudomonas* (representing 8% of all genera in NP_L_) includes species that are well known for their resistance to silver by the intracellular accumulation of Ag NPs [57].

Hitherto only limited studies have examined Ag NP toxicity on fungi, with the exception of yeast cultures [3], [58], [59]. Here we observed an increase in fungal groups by fatty acid analysis and changes in the fungal assemblage by DGGE and pyrosequencing. Helotiales, dominated by a *Chalara*, an anamorphic genus with saprotrophic and endotrophic species, declined in relative abundance by an order of magnitude from 75% in control soil to 7% after NP_L_ treatment. Representatives of the Hypocreales, however, showed an inverse trend by increasing from 1% in control to >70% of the fungal sequences. One of the Hypocreales, *Verticillium sp*., increased in abundance from <1% in controls to 14% after NP_L_-treatment (Fig. S2), and this genus is known for the intracellular synthesis of Ag NPs (review [60]). Another Hypocreales, the telomorph, *Cordyceps*, and its anamorph, *Isaria*, together dominated (∼50%) the nanosilver microcosms suggesting that these fungi have mechanisms that allow them to cope with the toxic effects of these particles. Indeed, these fungi are well known for their antioxidant properties [61], [62], [63]. Since a possible mode of NP-mediated toxicity is the generation of reactive oxygen species after membrane interaction [53], [64], [65], [66], [67], the antioxidant properties of these fungi may explain their ascendancy under these conditions.

### Conclusions

One of our goals was to investigate if concerns over the potential environmental impact of Ag NPs could simply be due to silver itself since this metal in bulk form has antimicrobial properties. Here we show that the size of the particle does matter; low concentrations of Ag NPs were more harmful than MPs, with toxicity unlikely due to ionic silver leaching since the NPs had an impact that was distinct from concentrations of MPs used to control for surface area. Further, we have shown that the addition of silver particles to soil coincident with temperature, fluctuations typical of a winter to summer transition, appears to interfere with the normal, temporal microbial changes in the arctic soil community, and with the potential to decrease soil fertility. Bacteria were generally more susceptible than fungi to these engineered NPs; likely this was due to the inability of the bacterial assemblage to recover quickly from temperature-regulated mitotic constraints in order to out-compete these eukaryotes for resources in the presence of Ag NPs. Alternatively, the arctic fungal assemblage could be more resistant. We have also shown that the plant-associating Rhizobiales was susceptible to NP toxicity but some bacterial species, including *Pseudomonas* and *Janthinobacterium*, as well as the fungal genera *Cordyceps*, and *Isaria* that are known for their antioxidant properties, appeared relatively resilient. Until we understand the possible long term impacts of the perturbed microbial community, we urge all nations to exert efforts not to deliberately contaminate the Arctic with such particles.

## Supporting Information

Figure S1 **Bacterial phylogenetic composition within the Daring Lake soil at the genus level.** Samples include soil that had been incubated for 86 days without NPs or MPs treatment (CS), soil incubated with 0.066% NPs (NP_L_), and soil with 0.066% MPs (MP_L_). Sequence identity was determined after pyrosequencing of the partial 16S rRNA genes, classified into genus, and the means of duplicate, replicate samples of those with >0.5% abundance (for either treatment or control groups) presented in separate categories, with groupings of less abundant genus shown as ‘Others’.
(TIF)

Figure S2 **Fungal phylogenetic composition within the Daring Lake soil at the genus level.** Samples include soil that had been incubated for 86 days without NPs or MPs (CS), soil incubated with 0.066% NPs (NP_L_), and soil with 0.066% MPs (MP_L_). Sequence identity was determined after pyrosequencing of the partial 18S rRNA genes, classified into genus, and the means of duplicate, replicate samples of those with >0.5% abundance (for either treatment or control groups) presented in separate categories, with groupings of less abundant genus shown as ‘Others’.
(TIF)
