# Supplementary figures and images for: The Effect of Silver Nanoparticles on Seasonal Change in Arctic Tundra Bacterial and Fungal Assemblages
Source: PLoS One. 2014 Jun 13;9(6):e99953. doi: 10.1371/journal.pone.0099953 (PMC4057283; doi:10.1371/journal.pone.0099953)

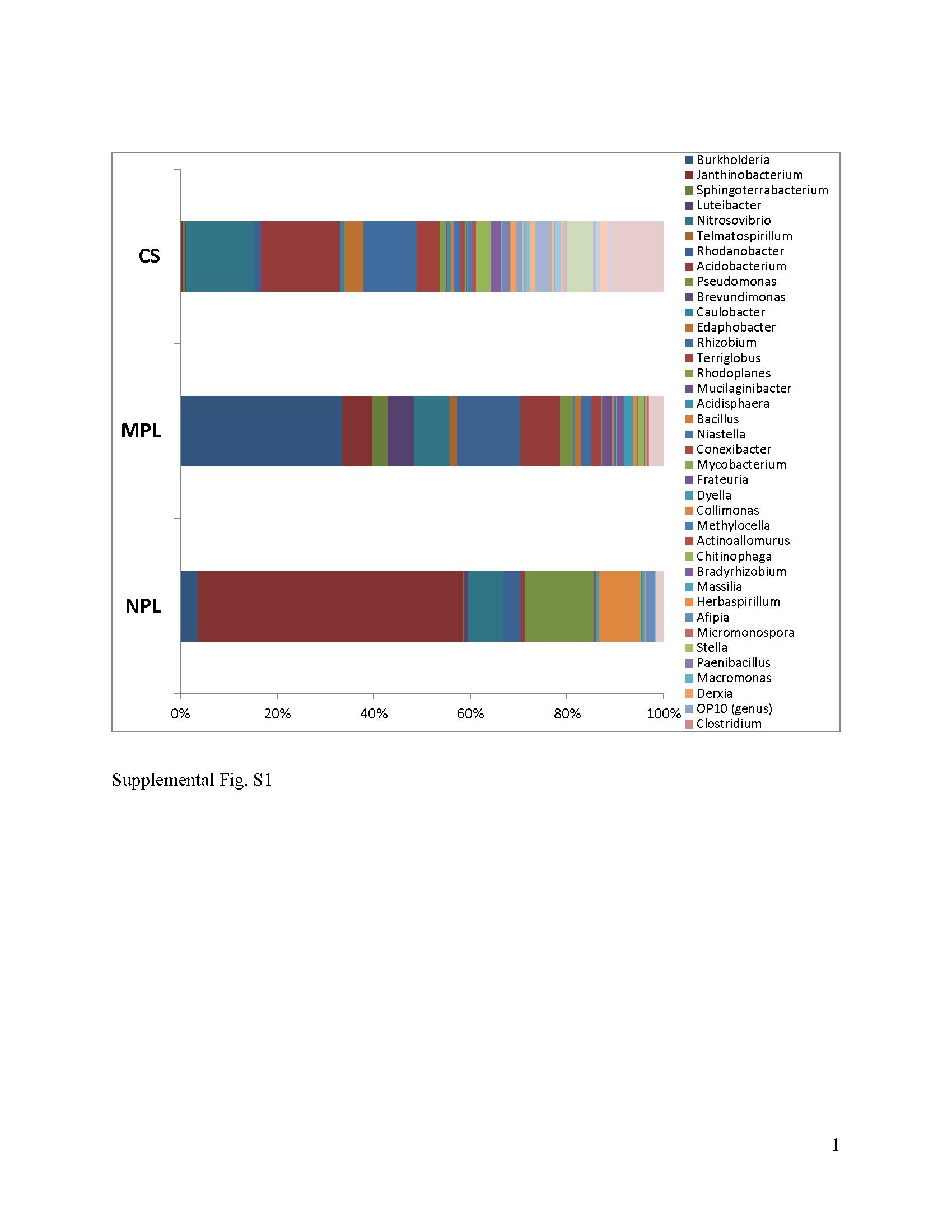

Supplement: Figure S1 — Bacterial phylogenetic composition within the Daring Lake soil at the genus level. Samples include soil that had been incubated for 86 days without NPs or MPs treatment (CS), soil incubated with 0.066% NPs (NPL), and soil with 0.066% MPs (MPL). Sequence identity was determined after pyrosequencing of the partial 16S rRNA genes, classified into genus, and the means of duplicate, replicate samples of those with >0.5% abundance (for either treatment or control groups) presented in separate categories, with groupings of less abundant genus shown as ‘Others’. (TIF) [file pone.0099953.s001.tif]

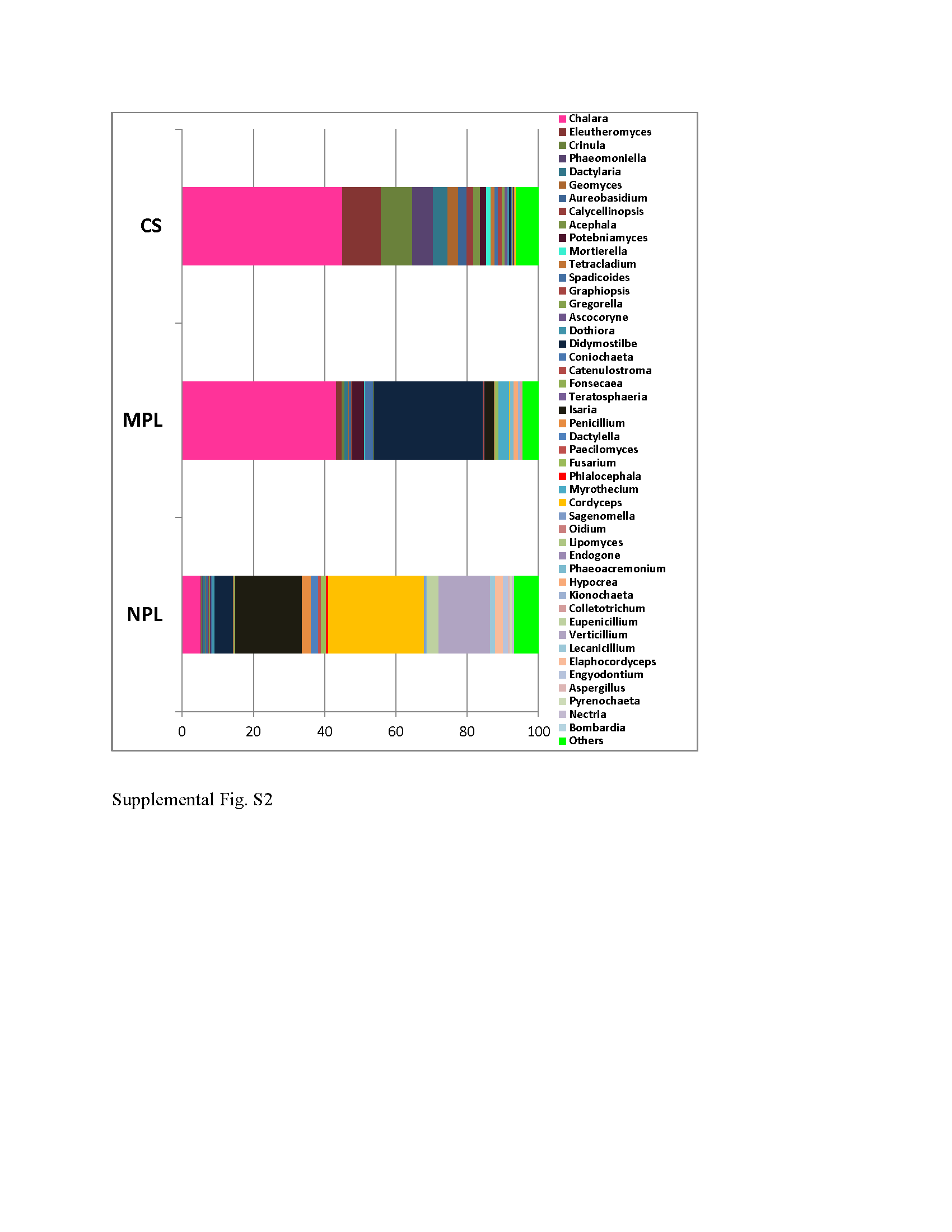

Supplement: Figure S2 — Fungal phylogenetic composition within the Daring Lake soil at the genus level. Samples include soil that had been incubated for 86 days without NPs or MPs (CS), soil incubated with 0.066% NPs (NPL), and soil with 0.066% MPs (MPL). Sequence identity was determined after pyrosequencing of the partial 18S rRNA genes, classified into genus, and the means of duplicate, replicate samples of those with >0.5% abundance (for either treatment or control groups) presented in separate categories, with groupings of less abundant genus shown as ‘Others’. (TIF) [file pone.0099953.s002.tif]
